# Microstructural Evolution and Mechanical Evaluation of a Laser-Induced Composite Coating on a Ni-Based Superalloy during Thermal Exposure

**DOI:** 10.3390/ma12091439

**Published:** 2019-05-03

**Authors:** Yuebing Li, Yanming He, Chuanyang Lu, Wenjian Zheng, Jianguo Yang, Donghong Wang, Limei Wang, Yuan Sun, Zengliang Gao

**Affiliations:** 1 Institute of Process Equipment and Control Engineering, Zhejiang University of Technology, Hangzhou 310014, China; ybli@zjut.edu.cn (Y.L.); lvcykk@163.com (C.L.); zwj0322@zjut.edu.cn (W.Z.); yangjg@zjut.edu.cn (J.Y.); lmwang@zjut.edu.cn (L.W.); zlgao@zjut.edu.cn (Z.G.); 2 Shanghai Key Lab of Advanced High-Temperature Materials and Precision Forming, School of Materials Science and Engineering, Shanghai Jiao Tong University, Shanghai 200240, China; wangdh2009@sjtu.edu.cn; 3 Department of Superalloy, Institute of Metal Research, Chinese Academy of Science, Shenyang 110016, China

**Keywords:** Ni-based superalloy, laser surface modification, thermal exposure, microstructure, mechanical property

## Abstract

A Ni–17Mo–7Cr-based superalloy was laser surface-modified to improve its tribological properties. Si particles were employed as coating materials. Si melted on the surface of the alloy during the process, triggering the formation of Mo_6_Ni_6_C carbides and Ni–Si intermetallics. A defect-free coating obtained was mostly made up of primary Mo_6_Ni_6_C and γ-Ni_31_Si_12_, as well as a eutectic structure of β_1_-Ni_3_Si and α-Ni-based solid solution (α-Ni (s.s)). The volume fraction of hard reinforcements (Mo_6_Ni_6_C, γ-Ni_31_Si_12_, and β_1_-Ni_3_Si) reached up to 85% in the coating. High-temperature microstructural stability of the coating was investigated by aging the coating at 1073 K for 240–480 h, to reveal its microstructural evolution. In addition, the mechanical performance of the coating was investigated. The nanoscale elastic modulus and hardness of Mo_6_Ni_6_C, γ-Ni_31_Si_12_, and α-Ni (s.s) were characterized using the nanoindentation tests. The nanoscratch tests were performed to measure the local wear resistance of the coating. Lastly, the Vickers hardness distribution across the cross-section of the coating before and after thermal exposure was compared. The work performed provides basic information understanding the microstructural evolution and mechanical performance of laser-induced coatings on Ni-based superalloys.

## 1. Introduction

Ni-based superalloys are widely used because of their excellent performances under harsh conditions [1]. There are four types of Ni-based superalloys: Monel (Ni–Cu-based), Inconel (Ni–Cr-based), Incoloy (Ni–Fe–Cr-based), and Hastelloy (Ni–Mo–Cr-based). The Ni–Mo–Cr-based Hastelloy alloys, manufactured by Haynes International, Inc, show outstanding high-temperature mechanical properties and strong corrosion resistances in reducing or oxidizing environments [2]. Many Hastelloy alloys (i.e., Hastelloy B [3], Hastelloy N [3], Hastelloy C276 [4], and Hastelloy X (HX) [5]) have been reported so far. The investigated Ni–17Mo–7Cr-based superalloy, namely Hastelloy N [6,7], was initially designed for use in nuclear industries. However, this kind of superalloy can also find applications in aerospace industries and chemical processing, as well as in oil and gas industries, and so forth [8,9,10,11]. In most cases, improving the wear resistance of materials is necessary to extend their service life. Some hard particles can be incorporated into the superficial layer of the substrate to form a composite material during laser surface modification, improving the wear and corrosion resistance [12,13]. These hard particles contain WC [14], SiC [15], Cr_3_C_2_ [16], Al_2_O_3_ [17], and so on. Apart from them, Mo_6_Ni_6_C carbides [18,19] and Ni–Si intermetallics [20,21] can also be imported as reinforcements. Wang et al. [18] reported that a laser-induced coating with the addition of Mo_6_Ni_6_C generated alloys with significantly higher hardness than that of the substrate. From the viewpoint of surface engineering, the Ni–Si intermetallics exhibited outstanding tribological properties due to their high hardness and strong atomic bonding [20]. Cai et al. [21] demonstrated that Ni–Si coating laser-cladded on carbon steel improved the wear and corrosion resistance.

We once introduced SiC particles as coating materials to produce a laser-induced coating on the Ni–17Mo–7Cr-based superalloy [22]. A crack-free coating containing widespread hard molybdenum carbides was achieved. Although lots of valuable results were obtained, many questions should be further studied. How to increase the volume fraction of hard phases in the coating is a big concern. It should be noted that the volume fraction of hard phases was merely 45.3% in the coating. Actually, the tribological ability of the coating was proportional to the amount of hard reinforcements incorporated. As expected, a higher quantity of reinforcements should produce an excellent wear resistance. In addition to that, microstructural stability of the laser-induced coating should be clarified at elevated temperatures. In general, a typical nonequilibrium solidified microstructure usually appears in the laser-induced coating, leading to undesirable phase transformations or serious elemental diffusions during thermal exposure, and then coating degradation [23].

The current investigation introduced Si particles as coating materials to produce a laser-induced layer on the Ni–17Mo–7Cr-based superalloy. Si rather than SiC was selected based on the following considerations: First, when SiC particles were used, they needed to be decomposed to take advantage of the triggering effect of Si [22]. The decomposition temperature of SiC reaches approximately 2873 K, whereas the melting point of Si is merely 1693 K. Compared with SiC, incorporating Si with a relatively lower melting point could lead to a cost-effective and easily controlled cladding procedure. Second, Si would melt with the surface layer of the alloy during the process, which would be beneficial to promote carbide precipitation [22,24]. Besides that, a great amount of Si would favor the precipitation of Ni–Si intermetallics. Both the carbides and Ni–Si intermetallics would act as hard reinforcements in the coating to resist wear [18,19,20,21,22,23]. Considering the fact that the amount of Si was adequate, it can be expected that an extremely high amount of hard reinforcements (over 45.3 vol.%) could be achieved in the coating, being a premise to achieve an excellent wear resistance. Lastly, in situ precipitation could generate a thermodynamically stable and compatible reinforcement/matrix interface [25].

For the laser-induced coating produced in the current investigation, detailed phase identification was performed and atomic contact at phase interface was investigated using transmission electron microscopy (TEM) and high-resolution transmission electron microscopy (HRTEM). In addition, the coating received was thermally exposed at 1073 K for 240–480 h to understand its microstructural stability. Lastly, the mechanical properties (nanoindentation, nanoscratch, and Vickers hardness tests) of the coating were evaluated. We focused on three aspects: (a) to provide a simple approach introducing a high amount of hard reinforcements in the coating, (b) to clarify the formation mechanism of the coating and elucidate its microstructural evolution during thermal exposure, and (c) to correlate the microstructure with mechanical performance for the coating.

## 2. Materials and Methods

The substrate used in the present investigation was a Ni–17Mo–7Cr-based superalloy with a Ni–17.29Mo–6.96Cr–3.96Fe–0.63Mn–0.47Si–0.05C (wt.%) composition, similar to that of Hastelloy N alloy. Before treatment, a layer (approximately 1 mm thick) of Si particles with an average diameter of 50 μm was coated on the substrate. A Leo HJ-3000 transverse-flow CO_2_ laser system was used to perform the laser surface modification with the parameters of laser power 2 kW, scanning speed 150 mm/min, and overlap ratio 50%. High-purity Ar was used to protect the molten pool from oxidation. The coating received was thermally exposed at 1073 K for 240 h and 480 h in a furnace with Ar protection. To examine the microstructure, the coating was mounted and prepared using a standard polishing procedure, followed by etching in a mixed solution (1 g FeCl_3_ + 10 mL HCl + 20 mL deionized water) for 1 min. The microstructure and crystallographic information of the nonaged and aged coating was studied using scanning electron microscopy (SEM, FEI Quanta 200F) and transmission electron microscopy (TEM, FEI Tecnai G2 F30). TEM foils were prepared by grinding followed by ion milling.

The triboindenter system (Hysitron Inc., Eden Prairie, MN, USA) coupled with a well-calibrated Berkovich tip was used to measure nanoscale elastic modulus (E) and hardness (H) of phases in the coating. A continuous stiffness measurement technique was selected [26]. The constitution of the investigated coating, as reported below, was rather complicated. Therefore, the nanoindentation measurements were performed on related phases rather than the entire coating. Before performing the tests, the coating was etched to reveal the phases. Before the tests, precise indent positions were preselected using the optical microscope the nanoindentation tester was equipped with. The optical microscope used had a maximum magnification of 1000×. To reduce the effect of the substrate, the maximal penetration depth was limited to 200 nm, according to the size/width of the phases. The distances between two adjacent indents should be larger than 100 μm to avoid interaction.

The local wear behaviors of the original alloy and coating were evaluated using a nanoscratch test, which was performed by applying a load increasing from 0 to 10 mN using a calibrated diamond tip. The scratch length and velocity were set to 500 μm and 10 μm/s, respectively. The scratch depth was recorded continuously during measurements and the scratch groove was captured using the optical microscope the tester was equipped with. Three repeats were performed for each condition.

The microhardness distribution across the cross-section of coating was determined using a Vickers hardness tester (SFMIT, HVS-1000M, Shanghai, China). A load of 50 g was used to reduce the effect of microstructural inhomogeneity. The testing points starting from the coating and moving toward the substrate had indent spacing of 50 μm.

## 3. Results and Discussion

### 3.1. Microstructure of the Coating before Thermal Exposure

**Figure 1:**
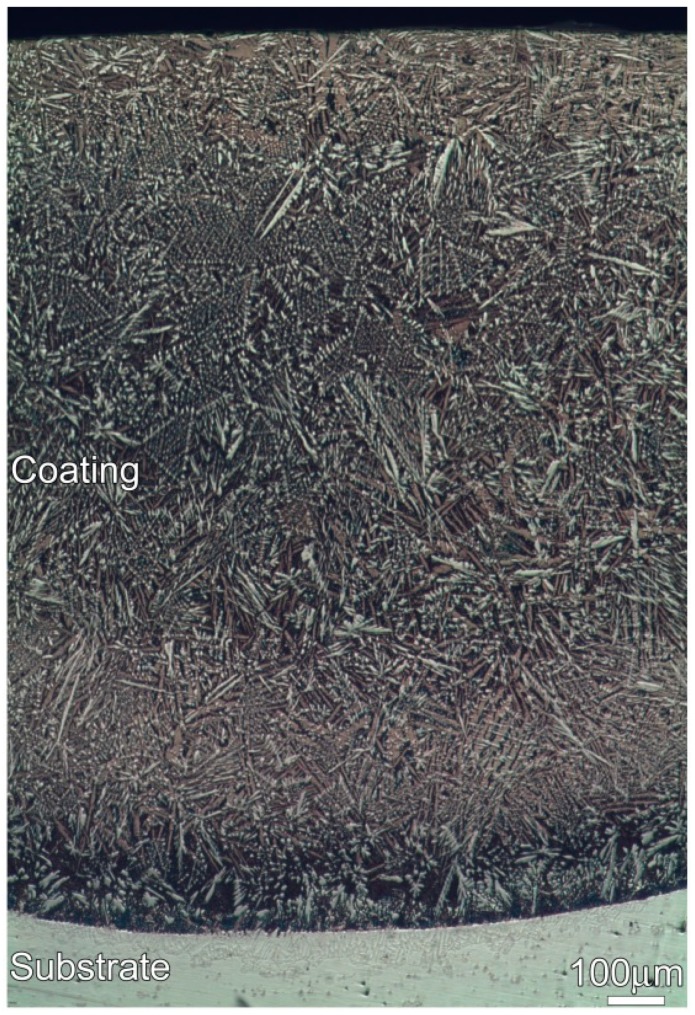
Optical metallograph of a cross-section of the coating.

**Figure 2:**
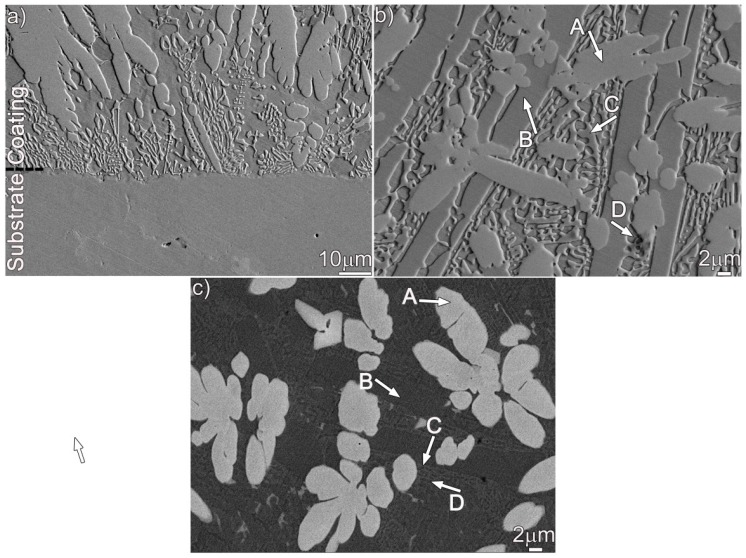
SEM secondary electron images (**a**,**b**) and backscattered electron image (**c**) showing the microstructure in the coating: (**a**) at the interface and (**b**,**c**) at the upper part.

Figure 1 shows the optical metallograph of a cross-section of the coating. An examination of the coating indicates that it has an average thickness of approximately 1.5 mm. No remarkable cracks or porosities can be found. Additionally, a good metallurgical bond is achieved at the coating/substrate interface. Figure 2 shows the SEM images of the cross-section of the Ni–17Mo–7Cr-based superalloy after laser surface modification. The microstructure of the coating consists of columnar and equiaxed dendrites with the columnar dendrites perpendicular to bonding interface. Four phases (A, B, C, and D) can be observed in the coating, as demonstrated in Figure 2b (secondary electron image) and Figure 2c (backscattered electron image). The four phases were evaluated using spot scan analysis to acquire their exact compositions, as shown in Table 1. Each value reported was averaged on at least three sets of data performed on the same phase. The elements Ni, Mo, Si, and C are enriched in phase A, while phase B and C mainly contain Ni and Si. For phase D, it primarily consists of Ni, Cr, Fe, and Si.

**Figure 3:**
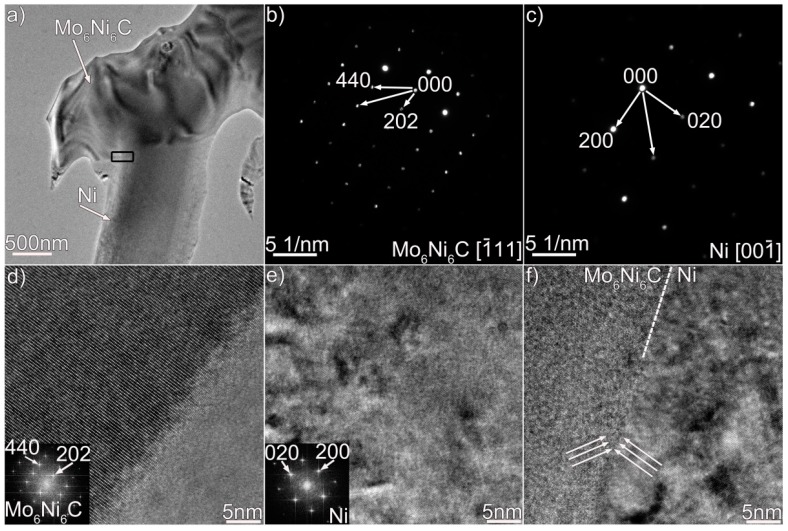
(**a**) Bright-field TEM micrograph of Mo_6_Ni_6_C and Ni in the coating, (**b**,**c**) SAED patterns of the Mo_6_Ni_6_C and Ni in (**a**), (**d**,**e**) HRTEM images of the Mo_6_Ni_6_C and Ni in (**a**), and (**f**) HRTEM image of the Mo_6_Ni_6_C/Ni interface enclosed in black box in (**a**).

**Figure 4:**
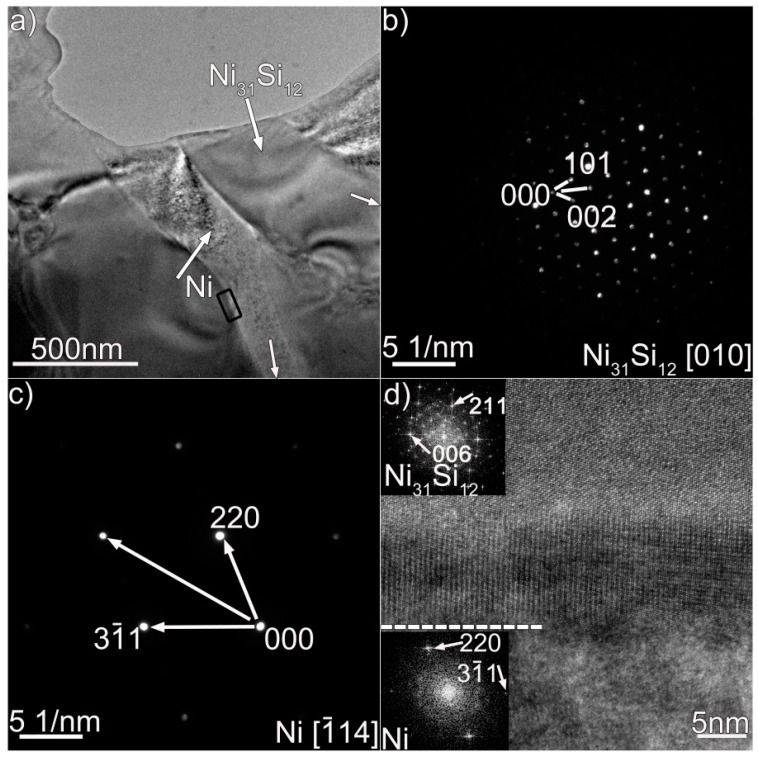
(**a**) Bright-field TEM micrographs of Ni_31_Si_12_ and Ni in the coating, (**b**,**c**) SAED patterns of the Ni_31_Si_12_ and Ni in (**a**), and (**d**) HRTEM image of the Ni_31_Si_12_/Ni interface enclosed in black box in (**a**).

**Figure 5:**
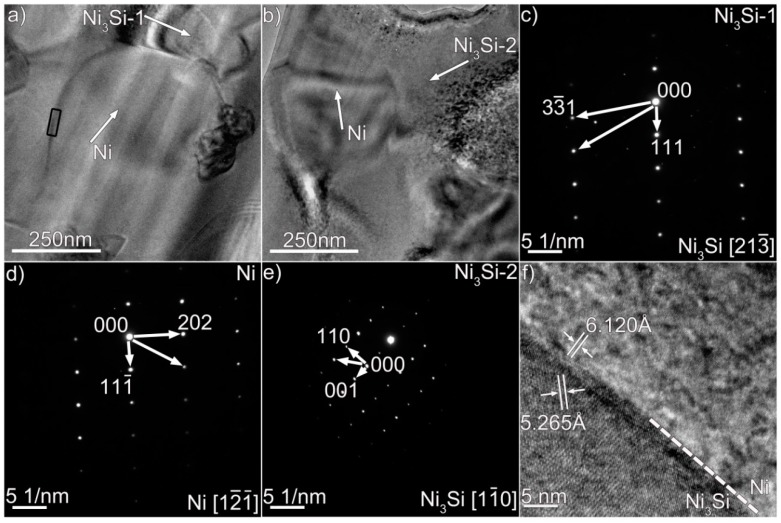
(**a**,**b**) Bright-field TEM micrographs of Ni_3_Si and Ni in the coating, (**c**,**d**) SAED patterns of the Ni_3_Si and Ni in (**a**), (**e**) SAED pattern of the Ni_3_Si in (**b**), and (**f**) HRTEM image of the Ni_3_Si/Ni interface enclosed in black box in (**a**).

We performed TEM analysis to clarify the nature of the coating, and the results are presented in Figure 3, Figure 4 and Figure 5, which contain properly indexed selected-area electron diffraction (SAED) pattern results. The Mo_6_Ni_6_C carbides, as shown in Figure 3a, are identified in the coating, based on the SAED and fast Fourier transformation (FFT) results in Figure 3b,d. The Mo_6_Ni_6_C is an M_6_C type of carbide (M represents metallic elements), having a cubic structure with a lattice constant of a = 10.891 Å. No coherency, as shown in Figure 3f, is noticed at the Mo_6_Ni_6_C/Ni interface along the investigated crystal plane. According to the SAED and HRTEM results in Figure 4 and Figure 5, two Ni–Si intermetallics, namely γ-Ni_31_Si_12_ and β_1_-Ni_3_Si, can be found in the coating. The γ-Ni_31_Si_12_ exhibits a hexagonal structure with lattice constants of a = 6.667 Å and c = 12.277 Å, whereas the β_1_-Ni_3_Si presents a cubic structure with lattice constant of a = 3.510 Å. In addition, cubic α-Ni with lattice constant of a = 3.535 Å can be detected, as illustrated in Figure 3c,e, Figure 4c, and Figure 5d. The Ni_31_Si_12_/Ni and Ni_3_Si/Ni interfaces (Figure 4d and Figure 5f) are free of defects, and no coherency is discovered. Briefly, four phases (Mo_6_Ni_6_C, γ-Ni_31_Si_12_, β_1_-Ni_3_Si, and α-Ni) were identified in the coating using TEM analysis. Coupled with the SEM/EDS results, phases A, B, C, and D in Figure 2b were therefore determined to be Mo_6_Ni_6_C, γ-Ni_31_Si_12_, β_1_-Ni_3_Si, and α-Ni, respectively.

According to Ref. [22], the carbides were simply identified as MoC in the coating based on the composition results. Electron diffraction analysis was not performed. In this investigation, Mo_6_Ni_6_C was identified based on the TEM and HRTEM results, particularly the diffraction pattern analysis shown in Figure 3b,d,f. These trustworthy TEM results strongly suggest that the carbides were essentially the Mo_6_Ni_6_C. The composition of MoC reported in Ref. [22] and that of Mo_6_Ni_6_C in this investigation presented discrepancies, particularly Si content (11 at.% Si for MoC and 20 at.% Si for Mo_6_Ni_6_C). It seemed that the different amounts of Si would induce different carbide types. Since this is a rather complicated problem which needs to be further investigated, relevant research will be carried out in future.

Based on the above analysis, a forming process of the coating was proposed: the surface of the alloy melted with Si particles when the laser beam scanned the surface of the alloy coated with Si particles. Afterward, the Si atoms were dissolved. The Mo_6_Ni_6_C columnar dendrites perpendicular to the contact interface were first formed at the bottom of the molten pool upon cooling, due to heat transfer through the substrate. At the top of the molten pool, the prevailing Si promoted the formation of Mo_6_Ni_6_C equiaxed dendrites. Many solute atoms were then expelled toward the interdendritic regions. The enrichment of Ni and Si atoms in the interdendritic regions facilitated the precipitation of lath-like Ni_31_Si_12_. According to the Ni–Si phase diagram [27], γ-Ni_31_Si_12_ was formed when the melt was cooled to 1515 K. A eutectic transformation took place in the restricted melt when the temperature cooled to 1416 K, producing α-Ni-based solid solution (α-Ni (s.s)) and β_3_-Ni_3_Si along with γ-Ni_31_Si_12_. Upon further cooling, the primary β_3_-Ni_3_Si underwent a polymorphic transformation (i.e., β_3_-Ni_3_Si→β_2_-Ni_3_Si) and a eutectoid transformation (i.e., β_3_-Ni_3_Si→β_1_-Ni_3_Si+γ-Ni_31_Si_12_). Both transformations were solid-state transformations, causing minor changes in the structure and composition of the crystal.

### 3.2. Microstructure of the Coating after Thermal Exposure

**Figure 6:**
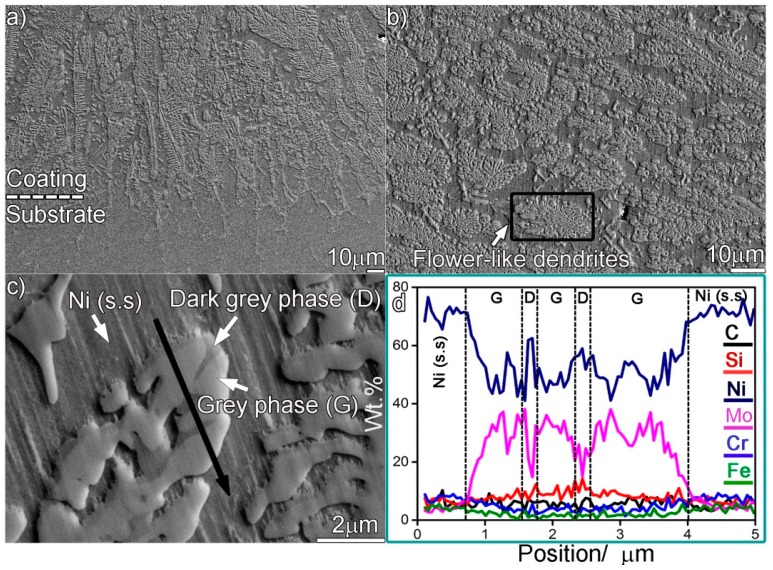
SEM secondary electron micrographs showing the microstructure in the coating aged for 240 h: (**a**) at the interface, (**b**) at the upper part, (**c**) magnified morphology of the upper part, and (**d**) elemental line scan analysis across black line in (**c**).

**Figure 7:**
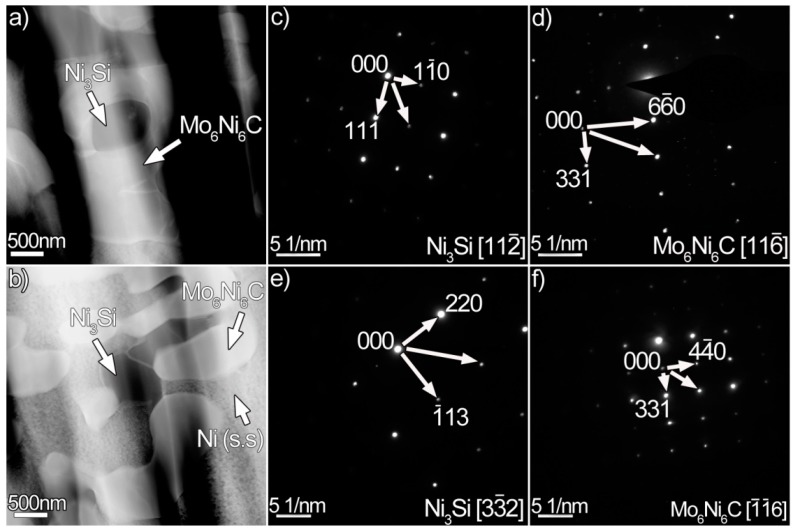
(**a**,**b**) High-angle annular dark field (HAADF) images of the coating aged for 240 h, (**c**,**d**) SAED patterns of the Ni_3_Si and Mo_6_Ni_6_C in (**a**), and (**e**,**f**) SAED patterns of the Ni_3_Si and Mo_6_Ni_6_C in (**b**).

The resulting microstructure of the laser-induced coating aged at 1073 K for 240 h is shown in Figure 6. An uneven coating/substrate interface rather than a smooth one, as indicated in Figure 6a, was formed. Many flower-like dendrites, consisting of round or nearly round particles, were distributed in the Ni (s.s) (Figure 6b). The detailed microstructure of these dendrites is illustrated in Figure 6c. It can be seen from the figure that the flower-like dendrites contain two phases: grey phase (G) and dark grey phase (D). The high enrichment in Ni (44 wt.%), Mo (32 wt.%), and Si (9 wt.%) in the grey phases signifies that they may be the Mo_6_Ni_6_C. When the scanning line contacts the dark grey phases (Figure 6d), the amounts of Ni and Si fluctuate toward their maximum values. The enrichment in Ni (79 wt.%) and Si (12 wt.%) indicate that the dark grey phases may be nickel silicides. Figure 7 presents the TEM results of the flower-like dendrites. The white phases in Figure 7a,b are identified as the Mo_6_Ni_6_C (Figure 7d,f). Some small particles are embedded in the Mo_6_Ni_6_C. Indexing the diffraction patterns demonstrated that these particles are actually the Ni_3_Si (Figure 7c,e). Thus, it can be concluded that the flower-like dendrites are actually mixtures of the Mo_6_Ni_6_C and nickel silicides. Associated with the original microstructure before thermal exposure, the occurrence of the flower-like dendrites suggests that the original Mo_6_Ni_6_C broke up, and nickel silicides transformed into Mo_6_Ni_6_C.

**Figure 8:**
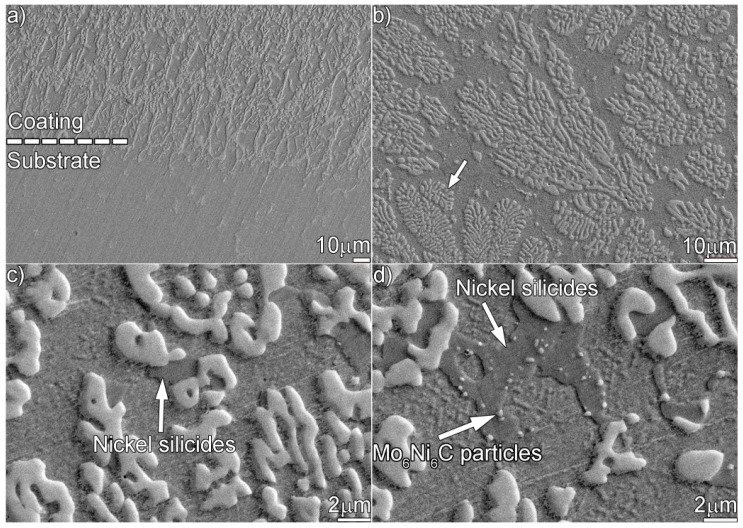
SEM secondary electron micrographs showing the microstructure in the coating aged for 480 h: (**a**) at the interface, (**b**) at the upper part, and (**c**,**d**) magnified morphology of the upper part.

**Figure 9:**
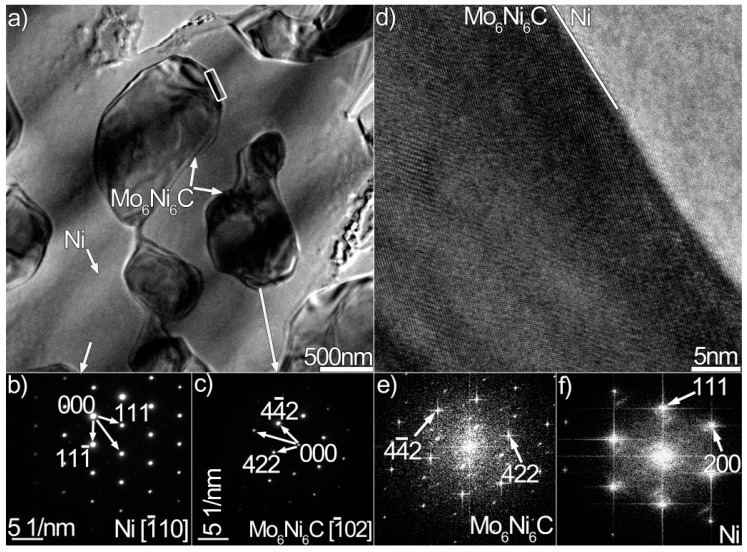
(**a**) Bright-field TEM micrograph of the coating aged for 480 h, (**b**,**c**) SAED patterns of the Ni and Mo_6_Ni_6_C in (**a**), (**d**) HRTEM image of the Mo_6_Ni_6_C/Ni interface enclosed in the white box in (**a**), and (**e**,**f**) FFT results of the Mo_6_Ni_6_C and Ni in (**d**).

Figure 8 shows the microstructure of the coating aged for 480 h. An irregular coating/substrate interface similar to the one obtained after 240 h is observed. It can be seen from Figure 8b that few flower-like dendrites (indicated by the white arrow) are observed, since a longer thermal exposure drove the original Mo_6_Ni_6_C dendrites to break up and become granulated further. Moreover, some residual nickel silicides, as indicated in Figure 8c,d, are still observed. Figure 9 shows the TEM results of the coating aged for 480 h. According to the SAED patterns in Figure 9b,c, the particles in Figure 9a are the Mo_6_Ni_6_C, while the matrix belongs to the Ni (s.s). In addition, a clean and defect-free Mo_6_Ni_6_C/Ni interface (Figure 9d) can be inspected.

The transformation of nickel silicides to carbides was rarely reported. In this investigation, two types of nickel silicides (i.e., γ-Ni_31_Si_12_ and β_1_-Ni_3_Si) took place in the nonaged coating. The melting temperatures of Ni_31_Si_12_ and Ni_3_Si were 1515 and 1416 K, respectively. Tasgin et al. [28] reported that the Ni_31_Si_12_ was precipitated in a white cast iron annealed at 1223 K. When a Ni/Si couple was annealed at 1173 K, the Ni_3_Si was reported to be generated [29]. Generally, the aging temperature of 1073 K used would not drive the transformation of nickel silicides to carbides. However, phase stability also depended on the elements incorporated. Because of a rapid solidification of the melt pool (103–1010 K/s) during laser treatment, the concentration of solute atoms in one phase often exceeded the solubility limit expected from equilibrium phase diagram. In addition to the main components, 57 at.% Ni and 26 at.% Si, the β_1_-Ni_3_Si under investigation contained 3 at.% Cr, 2 at.% Fe, and 12 at.% C. The element Cr was considered to have a beneficial effect on the Ni_3_Si, and Ni–Si alloy with Cr incorporation showed a significant strengthening effect over a wide range of temperatures [30]. The solubility of Fe should not exceed 5 at.% in the Ni_3_Si [31]. Himuro et al. [32] found that aging caused Ni_3_Si to transform into metastable (Ni, Fe)_3_Si in the Ni–Si–Fe sample with Fe content over 60 at.%. The carbon atoms were apt to be partitioned at the grain boundaries in the Ni_3_Si, suppressing intergranular fracture and improving ductility. However, its content should be kept below a certain value in the nickel silicides, 1 at.% in Ref. [33] and 0.15 at.% in Ref. [34]. Beyond that, precipitates with carbon enrichment or direct graphite precipitates would be formed. Therefore, thermal stability of the nickel silicides was actually controlled by the concentration of carbon. Up to 12 at.% C was identified in β_1_-Ni_3_Si after laser treatment. Ultra-supersaturation of carbon in the β_1_-Ni_3_Si and γ-Ni_31_Si_12_ was the critical reason for inducing their instability.

The transformation of the nickel silicides to Mo_6_Ni_6_C is summarized as below: during the initial stage of aging, the concentration fluctuation of carbon made the local nickel silicides (γ-Ni_31_Si_12_ and β_1_-Ni_3_Si) decompose, releasing Ni, Si, and C. The element Si was a critical element that would favor carbide precipitation [22,24]. Local enrichment in Ni and Si reacted with Mo and C, promoting the precipitation of tiny Mo_6_Ni_6_C particles. As demonstrated in Figure 8d, some nanosized Mo_6_Ni_6_C particles could be clearly detected in the nickel silicides. The main constituents of Mo_6_Ni_6_C were Mo, Ni, Si, and C, while small amounts of Cr and Fe were also incorporated. The decomposition of γ-Ni_31_Si_12_ or β_1_-Ni_3_Si provided sufficient Ni, Si, and C for the precipitation of Mo_6_Ni_6_C, while Mo was diffused from the surroundings. It can be inferred that the diffusion of Mo in nickel silicides was the critical process determining the precipitation rate of Mo_6_Ni_6_C.

The Mo_6_Ni_6_C dendrites, as mentioned, broke up and became granulated during thermal exposure. Compared with the original Mo_6_Ni_6_C, the concentration of Mo increased in the aged Mo_6_Ni_6_C (480 h), while the content of Si, Cr, and Fe decreased (Mo33 → 40 wt.%, Si11 → 8 wt.%, Cr6 → 4 wt.%, and Fe2 → 1 wt.%). Thermodynamic data predicted that M_6_C remained stable below 1573 K [35]. Therefore, the Mo_6_Ni_6_C under investigation would not degrade at 1073 K. However, carbides could tie up certain elements that facilitated their instability when aging or during service. The transformation of M_6_C to M_23_C_6_ was reported [36]. Considering the composition results, the deficiency of Mo in the original Mo_6_Ni_6_C should be the critical reason that induced their instability. The rapid cooling rate (10^3^–10^10^ K/s) caused many solute atoms to be detained in the original Mo_6_Ni_6_C during laser treatment, which would decrease the amount of Mo. Thus, the Mo-deficient Mo_6_Ni_6_C dendrites were actually produced. The Mo-deficient Mo_6_Ni_6_C tended to break up during aging, but the breaking sequence presented divergency at different positions, being related to the unhomogeneity of microchemical concentration. Upon cooling, a large temperature gradient would produce Mo_6_Ni_6_C dendrites with diverse surface curvatures. According to the Gibbs–Thomson law [37], the solute concentration at different positions in the dendrites could be expressed as:

(1) $$C_{\alpha }\left(r\right)=C_{\alpha }\left(\infty \right)\exp\left(\frac{\sigma V_{c}}{k_{c}Tr}\right)$$

where *C_α_*(*r*) is the solute concentration at curvature radius *r*, *C_α_*(∞) is the solute concentration at the plane interface, *σ* is the interfacial tension, *V_c_* is the volume of the solute atoms, *T* is the temperature, and *K_c_* is the shape coefficient. It can be seen from the equation that the smaller *r* was, the greater *C_α_*(*r*) would be. Therefore, the dendrites with smaller curvatures tended to be enriched with more solute atoms. High-temperature thermal exposure enhanced elemental diffusion. Therefore, the roots of the secondary or tertiary dendrite arms would first break up. As demonstrated in Figure 6b, a significant number of flower-like Mo_6_Ni_6_C dendrites were detected when aging the coating for 240 h. During the process, many solute atoms were released and redistributed. Therefore, the concentration of solute atoms (i.e., Si, Cr, and Fe) decreased in the aged Mo_6_Ni_6_C, while the content of Mo increased. Prolonged thermal exposure would promote more secondary, tertiary, or primary Mo_6_Ni_6_C dendrites breaking up. No bulk Mo_6_Ni_6_C dendrites were observed when aging the coating for 480 h. The Mo_6_Ni_6_C particles, as mentioned, had a tendency to become granulated. The granulation was actually a spontaneous process, leading to the reduction of the interfacial energy. Based on the above analysis, high-temperature thermal exposure promoted the transformation of nickel silicides (γ-Ni_31_Si_12_ and β_1_-Ni_3_Si) to Mo_6_Ni_6_C, and also caused the original Mo_6_Ni_6_C to break up and become granulated.

### 3.3. Mechanical Behavior of the Coating before and after Thermal Exposure

As mentioned, the laser-induced coating under investigation mostly consisted of the primary Mo_6_Ni_6_C and γ-Ni_31_Si_12_, as well as a eutectic structure of β_1_-Ni_3_Si and α-Ni (s.s). Among them, the Mo_6_Ni_6_C was carbide, while the γ-Ni_31_Si_12_ and β_1_-Ni_3_Si belonged to intermetallics. As shown in Figure 2b,c, many of the Mo_6_Ni_6_C and γ-Ni_31_Si_12_ particles were wider than 10 μm. Some of the α-Ni (s.s) particles were also wider than 10 μm. However, most of the β_1_-Ni_3_Si particles were restrained by the γ-Ni_31_Si_12_, presenting widths smaller than 2 μm. Therefore, the nanoindentation tests were performed on the Mo_6_Ni_6_C, γ-Ni_31_Si_12_, and α-Ni (s.s), rather than the β_1_-Ni_3_Si. In addition, nanoscale elastic modulus (E) and hardness (H) of Mo_6_Ni_6_C, γ-Ni_31_Si_12_, and α-Ni (s.s) were measured by depth-sensing indentation.

**Figure 10:**
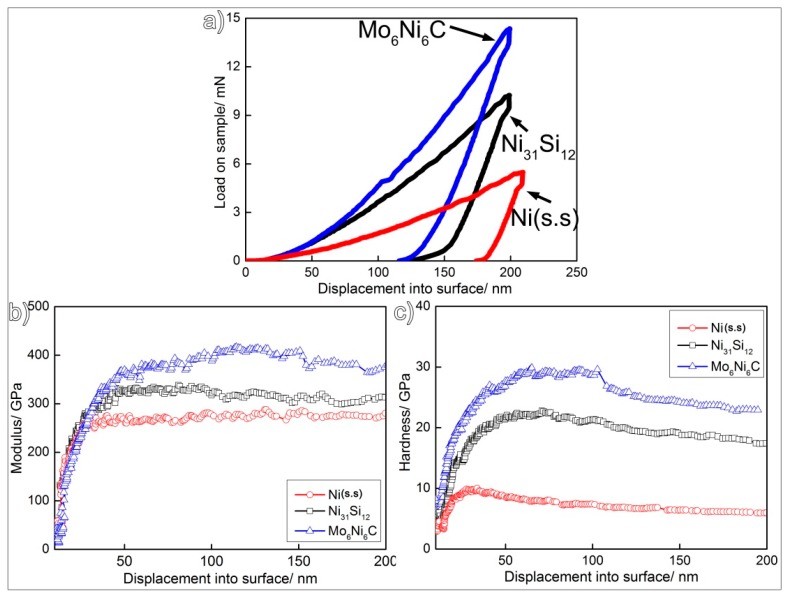
Nanoindentation results of (**a**) load–displacement curve, (**b**) indentation modulus–displacement curve, and (**c**) hardness–displacement curve for the Mo_6_Ni_6_C, γ-Ni_31_Si_12_, and α-Ni (s.s).

Figure 10a shows the representative load–displacement curves obtained. It can be seen that a higher indentation load is required for Mo_6_Ni_6_C and γ-Ni_31_Si_12_ to achieve the same penetration depth compared to α-Ni (s.s), suggesting that the Mo_6_Ni_6_C and γ-Ni_31_Si_12_ have higher hardness values than α-Ni (s.s). As can be seen in Figure 10b, the indentation moduli of the three phases (Mo_6_Ni_6_C, γ-Ni_31_Si_12_, and α-Ni (s.s)) increased rapidly in the 0–50 nm depth range, and then stabilized when the depth was over 50 nm. The hardness profiles also exhibited similar trends (Figure 10c). The average E and H values for the Mo_6_Ni_6_C, γ-Ni_31_Si_12_, and α-Ni (s.s) were 399.6 and 24.9, 327.2 and 19.6, and 275.6 and 6.1 GPa, respectively.

**Figure 11:**
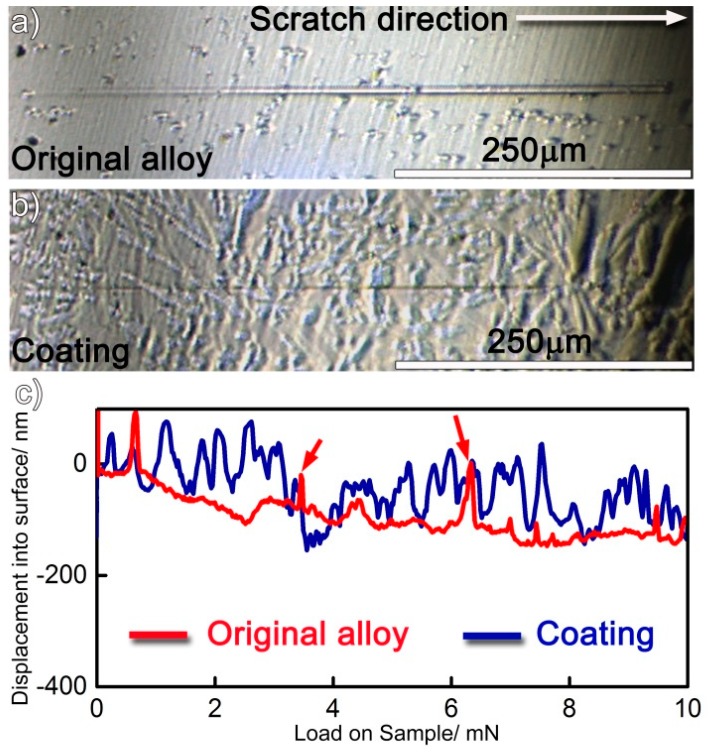
(**a**,**b**) Nanoscratch impressions on the original alloy and coating, and (**c**) scratch depth profile vs normal applied load curves.

The coating, as mentioned, contained Mo_6_Ni_6_C, γ-Ni_31_Si_12_, β_1_-Ni_3_Si, and α-Ni (s.s). Compared with the α-Ni (s.s), the Mo_6_Ni_6_C, γ-Ni_31_Si_12_, and β_1_-Ni_3_Si were harder, as demonstrated from the nanoindentation results. The nanoscratch measurement was performed with the aim to differentiate the effect of soft and hard phases in resisting local wear. The original alloy was also tested for comparison. The samples performed by the measurement were prepared under the same condition (just polishing without etching) to maintain consistency. The same preparation ensured the scratch curve was determined by the constitution of sample rather than surface topography. Figure 11a,b shows representative residual grooves. As expected, the residual scratch groove on the coating is always shallower and narrower than that on the original alloy under the same load, suggesting that the coating exhibits superior local scratch resistance compared with the original alloy. Penetration depth vs load-on-sample curves are presented in Figure 11c. For the original alloy, it is evident that the scratch penetration depth increased as the normal load was increased from 0 to 10 mN. Particularly, the depth, as indicated by red arrows in Figure 11c, fluctuated in some positions. Moreover, it should be noted that the original alloy basically consisted of Ni (s.s) and molybdenum carbides. When the scratch tip approached the hard carbides during scratching, the depth fluctuated. Compared with the original alloy, the penetration depth fluctuated more frequently on the coating. As noted, the Mo_6_Ni_6_C, γ-Ni_31_Si_12_, and β_1_-Ni_3_Si belonged to hard phases in the coating. In order to obtain their volume fraction in the coating, at least three SEM images with a low magnification (500×) were selected and each phase was identified in the figures. After that, the volume fraction of each phase was determined in the coating by making statistics. Each value reported was averaged for at least three sets of data. Quantitative analysis of the data indicated that the coating consisted of 30 vol.% Mo_6_Ni_6_C, 43 vol.% γ-Ni_31_Si_12_, 12 vol.% β_1_-Ni_3_Si, and 15 vol.% α-Ni (s.s). Therefore, laser surface modification imported up to 85 vol.% hard phases into the coating. Therefore, the penetration depth fluctuated frequently when the scratch load was performed on the coating. 

The frequent fluctuation during nanoscratch tests also suggests that the coating had an excellent local wear resistance, also being related with the coating obtained free of cracks and pores (see Figure 1 and Figure 2). The Mo_6_Ni_6_C, γ-Ni_31_Si_12_, and β_1_-Ni_3_Si phases were grown directly from the melt pool, and no discontinuities were detected at the interface (Figure 3, Figure 4 and Figure 5). Therefore, a strong interfacial bonding was considered to be achieved, precluding the longitudinal cracks from the coating. Cracks passing through reinforcements (i.e., WC) might also take place in the laser-induced coating, because most of the reinforcements presented low plastic deformability [38]. To eliminate such a crack, hard reinforcements incorporated should have a certain ability of plastic deformation. In this investigation, the hard reinforcements were actually the Mo_6_Ni_6_C, γ-Ni_31_Si_12_, and β_1_-Ni_3_Si. The nickel silicides (γ-Ni_31_Si_12_ and β_1_-Ni_3_Si) exhibited excellent high-temperature oxidation resistance and high hardness [39]. The poor ductility and lack of fabricability, however, limited their use. The intrinsic reason for the poor ductility and lack of fabricability was ascribed to grain-boundary embrittlement, particularly hydrogen embrittlement at room temperature and oxygen embrittlement at approximately 873 K [40]. Generally, the embrittlement could be ameliorated by microalloying treatment. During laser surface modification, the solidification of coating was a typical nonequilibrium process, which could cause the retention of many solute atoms far beyond their solubility limit. Except for Ni and Si, β_1_-Ni_3_Si also contained 4 wt.% C and 2 wt.% Cr. Incorporating C and Cr into nickel silicides improved their ductility [41]. Therefore, improving the ductility of Mo_6_Ni_6_C, γ-Ni_31_Si_12_, and β_1_-Ni_3_Si due to the microalloying was also critical to eliminate cracks from the coating obtained.

**Figure 12:**
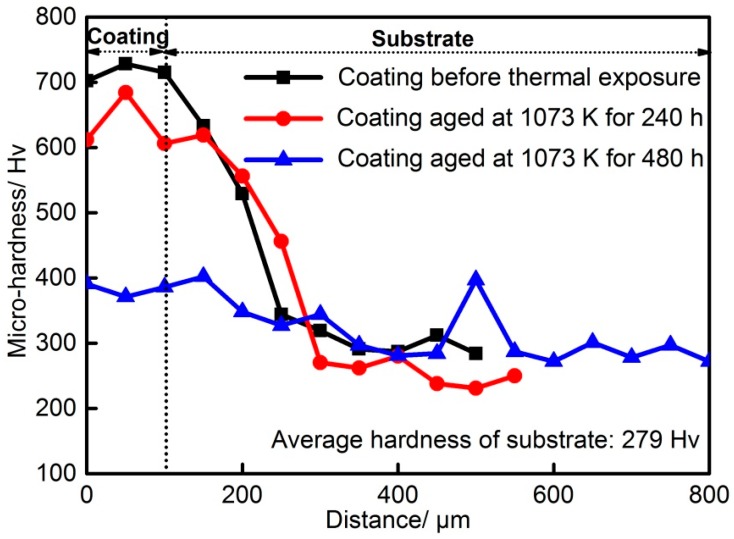
Vickers hardness distribution across the cross-section of the nonaged and aged coatings.

Figure 12 shows the results of Vickers microhardness distribution across the cross-section of the nonaged and aged coatings. As revealed, the hardness of the coating is always higher than that of the substrate, irrespective of the treatment of thermal exposure for the coating. In addition, the hardness of the coating decreases with increasing aging time. When the coating was aged for 480 h, its hardness was halved, compared to the hardness of the nonaged coating. As mentioned above, the coating contained a large amount of hard phases (Mo_6_Ni_6_C, γ-Ni_31_Si_12_, and β_1_-Ni_3_Si), contributing to the higher hardness of the nonaged coating. Thermal exposure made Mo_6_Ni_6_C become granulated and nickel silicides decompose, which declined the volume fraction of the hard phases, as has been demonstrated in Figure 2, Figure 6, and Figure 8.

## 4. Conclusions

The current investigation employed a simple way of importing a large amount of reinforcements (up to 85 vol.%) into the laser-induced coating generated on the surface of the Ni–17Mo–7Cr-based superalloy. Mechanical characterization tests (nanoindentation, nanoscratch, and Vickers hardness tests) were performed on the coating. Afterward, stability of coating was evaluated during high-temperature thermal exposure. The following conclusions were drawn:

(1) The novel approach involved took advantage of the triggering effect of Si in promoting the precipitation of Mo carbides and Ni–Si intermetallics. The coating obtained primarily consisted of the Mo_6_Ni_6_C, γ-Ni_31_Si_12_, and a mixture of α-Ni (s.s) and β_1_-Ni_3_Si eutectics. The volume fraction of the hard phases (Mo_6_Ni_6_C, γ-Ni_31_Si_12_, and β_1_-Ni_3_Si) reached up to 85%.
(2) High-temperature (1073 K) thermal exposure promoted the transformation of nickel silicides (γ-Ni_31_Si_12_ and β_1_-Ni_3_Si) to Mo_6_Ni_6_C. The supersaturation of carbon in the nickel silicides was the critical reason for inducing their instability. In addition, the deficiency of Mo in the original Mo_6_Ni_6_C caused them to break up and become granulated.
(3) We found E and H for the Mo_6_Ni_6_C, γ-Ni_31_Si_12_, and α-Ni (s.s) to be 399.6 and 24.9, 327.2 and 19.6, and 275.6 and 6.1 GPa, respectively.
(4) The nanoscratch tests indicated that the hard phases (Mo_6_Ni_6_C, γ-Ni_31_Si_12_, and β_1_-Ni_3_Si) in the coating had a strong ability to resist local wear.
(5) The Vickers microhardness values of the nonaged and aged coatings were always higher than that of the substrate. Thermal exposure, however, lowered the hardness of the coating, compared to the nontreated one.

The results performed can be applied not only to the Ni–17Mo–7Cr-based Hastelloy N alloy, but also to other Hastelloy series alloys that require a wear-resistant coating. Friction and wear tests will be performed for the laser-induced coatings before and after thermal exposure in the near future.

## Figures and Tables

**Table 1:** Chemical composition of different phases in the coating.

Phase	Composition (wt.%)					
	C	Si	Ni	Mo	Cr	Fe
A	5	11	43	33	6	2
B	4	15	76	1	2	2
C	4	16	74	1	3	2
D	4	7	65	2	13	9
